# Effects of lactate administration on hypertrophy and mTOR signaling activation in mouse skeletal muscle

**DOI:** 10.14814/phy2.15436

**Published:** 2022-08-22

**Authors:** Takanaga Shirai, Yu Kitaoka, Kazuki Uemichi, Katsuyuki Tokinoya, Kohei Takeda, Tohru Takemasa

**Affiliations:** ^1^ Faculty of Health and Sport Sciences University of Tsukuba Tsukuba Ibaraki Japan; ^2^ Research Fellow of Japan Society for Promotion Science Chiyoda‐ku Tokyo Japan; ^3^ Department of Human Sciences Kanagawa University Yokohama‐shi Kanagawa Japan; ^4^ Graduate School of Comprehensive Human Sciences University of Tsukuba Tsukuba Ibaraki Japan; ^5^ Division of Clinical Medicine, Faculty of Medicine University of Tsukuba Tsukuba Ibaraki Japan; ^6^ Department of Health Promotion Sciences Graduate School of Human Health Sciences Tokyo Metropolitan University Hachioji Tokyo Japan; ^7^ School of Political Science and Economics Meiji University Suginami‐ku Tokyo Japan

**Keywords:** hypertrophy, lactate, mTOR signaling, protein synthesis, skeletal muscle

## Abstract

Lactate is a metabolic product of glycolysis and has recently been shown to act as a signaling molecule that induces adaptations in oxidative metabolism. In this study, we investigated whether lactate administration enhanced muscle hypertrophy and protein synthesis responses during resistance exercise in animal models. We used male ICR mice (7–8 weeks old) were used for chronic (mechanical overload induced by synergist ablation: [OL]) and acute (high‐intensity muscle contraction by electrical stimulation: [ES]) resistance exercise models. The animals were intraperitoneally administrated a single dose of sodium lactate (1 g/kg of body weight) in the ES study, and once a day for 14 consecutive days in the OL study. Two weeks of mechanical overload increased plantaris muscle wet weight (main effect of OL: *p* < 0.05) and fiber cross‐sectional area (main effect of OL: *p* < 0.05), but those were not affected by lactate administration. Following the acute resistance exercise by ES, protein synthesis and phosphorylation of p70 S6 kinase and ribosomal protein S6, which are downstream molecules in the anabolic signaling cascade, were increased (main effect of ES: *p* < 0.05), but lactate administration had no effect. This study demonstrated that exogenous lactate administration has little effect on the muscle hypertrophic response during resistance exercise using acute ES and chronic OL models. Our results do not support the hypothesis that elevated blood lactate concentration induces protein synthesis responses in skeletal muscle.

## Graphical abstract

In this study, we examined the effects of lactate administration on the activation of exercise‐induced protein synthesis signals and muscle hypertrophy using acute and chronic resistance exercise models. We found no effect of lactate on the acute or chronic exercise models with respect to hypertrophy and exercise‐induced mTORC1 signaling."

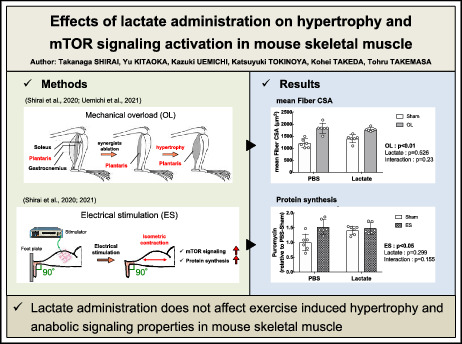

## 1 INTRODUCTION

Skeletal muscle mass is defined by the balance between muscle protein synthesis and degradation, The anabolic condition, in which protein synthesis exceeds degradation, therefore induces muscle hypertrophy (Miyazaki & Esser, 2009). Resistance exercise (e.g., high‐intensity muscle contractions, mechanical overload) promotes muscle protein synthesis and fiber hypertrophy (Damas et al., 2016; Dickinson et al., 2011). The molecular mechanism by which resistance exercise promotes skeletal muscle hypertrophy is not entirely understood, but the mechanistic target of rapamycin (mTOR) signaling has been reported to significantly contribute to physiological process (Bodine et al., 2001; Rommel et al., 2001). Resistance exercise enhances phosphorylation of p70 S6 kinase (p70S6K), ribosomal protein S6 (S6), and eukaryotic initiation factor 4E‐binding protein 1 (4E‐BP1), which are canonical downstream signals of mTOR, and it increases the efficiency of gene translation (Ogasawara et al., 2016).

Resistance exercise significantly decrease muscle glycogen content and increases glycolytic metabolites; however, glycolysis inhibition attenuates acute resistance exercise‐induced increases in mTOR signaling activity and muscle protein synthesis (Suginohara et al., 2021). Moreover, low‐load resistance exercise with blood flow restriction, which increases blood lactate levels, has been shown to be effective for increasing muscle mass, (Yoshikawa et al., 2019) suggesting that glycolytic metabolites might contribute to muscle hypertrophy. Lactate, a product of the glycolytic system, has been regarded as a mere waste product; in recent years, however, it has been recognized as a potential signaling molecule that induces beneficial adaptations in various tissues (Brooks, 2018; Ferguson et al., 2018). In skeletal muscle, lactate has been reported to not only to increase mitochondrial enzyme activity (Takahashi et al., 2019) but also to have a hypertrophic effect through C2C12 cells (Ohno et al., 2018). A previous study has reported that in vivo lactate administration also activates anabolic intracellular signaling pathways in mature mouse skeletal muscle (Cerda‐Kohler et al., 2018). However, it is still unclear whether lactate administration enhances resistance exercise‐induced muscle protein synthesis and subsequent hypertrophy. Therefore, this study aimed to determine the effects of lactate on the muscle hypertrophy induced by chronic mechanical overload and the protein synthesis response induced by acute high‐intensity muscle contractions.

## 2 MATERIAL METHODS

### 2.1 Animals

**FIGURE 1:**
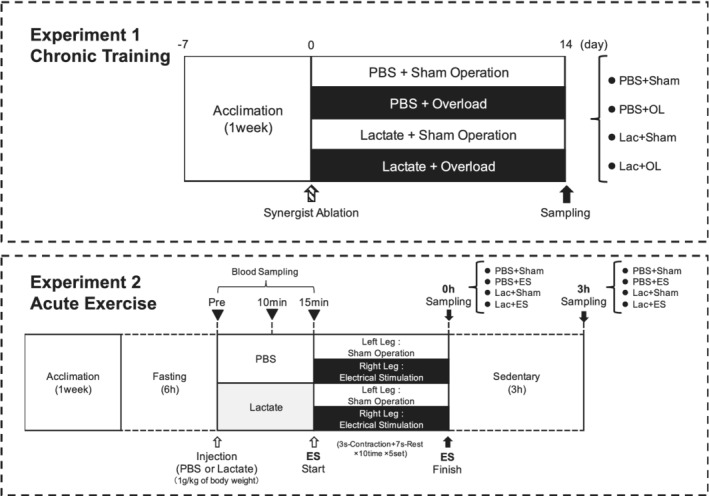
Study designs

All experimental procedures performed in this study were approved by the Institutional Animal Experiment Committee of the University of Tsukuba (animal ethical approval number: 20–407) based on the National Institutes of Health guidelines for the Care and Use of Laboratory Animals (NIH publication, 1996). Male ICR mice aged 7–8 weeks (Tokyo Laboratory Animals Science Co., Tokyo, Japan) were used in this study. Mice were kept in temperature (22 ± 2°C) and humidity (55 ± 5%)‐controlled facilities under a 12/12‐h light/dark cycle with ad libitum access to food and water. The experimental protocol is shown in Figure 1.

### 2.2 Experiment 1: Lactate administration during chronic mechanical overload

We performed lateral synergist ablation surgeries, as previously described, (Shirai, Obara, & Takemasa, 2020; Uemichi et al., 2021) under anesthesia with 2.0% isoflurane air inhalation. This in vivo model induces hypertrophy of the plantaris muscle by mechanical overload (OL), through the surgical removal of synergist muscles (gastrocnemius and soleus) and is very useful as a model to observe exercise‐induced muscle hypertrophy and protein synthesis (Moriya & Miyazaki, 2018; Uemichi et al., 2021). One of the characteristics of the plantaris muscles used in this experiment is that the type IIA or IIB fibers are dominant (Miyazaki et al., 2004; Souza et al., 2014). The mice were divided 4 groups: PBS‐administrated sham operation group (PBS + Sham; *n* = 6), PBS‐administrated OL group (PBS + OL; *n* = 6), lactate‐administrated sham operation group (Lac + Sham; *n* = 6), lactate‐administrated OL group (Lac + OL; *n* = 6). The mice were intraperitoneally administrated phosphate buffered saline (PBS) or 1 g/kg of body weight of sodium lactate once a day at 12:00 for 14 consecutive days. Fourteen days after surgery, the mice were anesthetized, and the plantaris muscle was excised, weighed, quickly frozen in liquid nitrogen, and stored at −80°C until needed for analysis.

### 2.3 Experiment 2: Lactate administration following acute electrical muscle stimulation

The resistance exercise protocol was conducted as previously described (Ogasawara et al., 2016; Shirai, Aoki, et al., 2020). Briefly, the mice were anesthetized with inhaled isoflurane (2%, KN‐1701; Natsume), and both lower limbs of each mouse were shaved and cleaned with alcohol wipes. Lactate or PBS was then administrated intraperitoneally 15 min before the resistance exercise. The mice were positioned with their foot on a footplate (with an ankle joint angle of 90°) in the prone position. The gastrocnemius muscle was stimulated percutaneously with electrodes connected to an electric stimulator and isolator (Ag/AgCl, Vitrode V; Nihon Kohden). The right gastrocnemius muscle was isometrically exercised (stimulation for 3 s, 10 contractions, with intervals of 7 s between contractions; total of 5 sets with 3‐min intervals between sets). The voltage (30 V) and stimulation frequency (100 Hz) were adjusted to produce maximal isometric tension. The left gastrocnemius muscle served as a control (Sham), and electrodes were attached, but no ES was applied. Blood samples were collected from the tail before and 15 min after administrating lactate or PBS. Blood lactate levels were measured using a portable blood lactate analyzer (Lactate Pro 2, Arkray). Muscle samples were obtained immediately (0 h) and 3 h after resistance exercise. Tissues were frozen rapidly in liquid nitrogen and stored at −80°C until use. We have previously confirmed that electrical stimulation causes contraction of the gastrocnemius muscle, but the effect on muscles such as soleus and plantaris is unknown, so we sampled the gastrocnemius muscle. The muscle fibers of the gastrocnemius muscle used in this experiment are characterized by Type II and IIB fibers (Rahmati & Rashno, 2021; Vechetti et al., 2019).

### 2.4 Lactate tolerance test

The resistance exercise protocol was conducted as previously described (Takahashi et al., 2020; Takahashi et al., 2021). The lactate tolerance test was conducted after intraperitoneal administration of sodium lactate (1 g/kg body weight) 14 days after the start of OL. Blood samples were taken from the tail before and 5, 15, 30, and 60 min after administration. Blood lactate concentration was measured using a portable blood lactate analyzer (Lactate Pro 2, Arkray).

### 2.5 Cross sectional area quantification

The plantaris muscle was covered optimal cutting temperature (OCT) compound (Sakura Finetek), rapidly frozen in liquid nitrogen‐cooled isopentane, and stored at −20°C until sectioning. Frozen muscles were sectioned at a thickness of 10 μm, air dried, and stored at −20°C. Sections were fixed in 4% paraformaldehyde (PFA), permeabilized with 0.1% Triton X‐100, and blocked with 1% bovine serum albumin. Rat anti‐laminin and Texas Red‐conjugated goat anti‐rat immunoglobulin G (H + L) antibodies were used for detecting laminin localization. The CSA of muscle fiber sections was measured using laminin‐stained 20× magnification images. Image were captured with an Olympus DP‐74 microscope (Tokyo, Japan), and the CSA analysis was performed using the Image J software.

### 2.6 Western blotting

The excised plantaris and gastrocnemius muscles were immediately frozen in liquid nitrogen, and total muscle protein was extracted using lysis buffer containing 50 mM HEPES (pH: 7.6), 150 mM NaCl, 10 mM EDTA, 10 mM Na_4_P_2_O_7_, 10 mM NaF, 2 mM Na_3_VO_4_, 1% (vol/vol) NP‐40, 1% (vol/vol) Na‐deoxycholate, 0.2% (wt/vol) sodium dodecyl sulphate, and 1% (vol/vol) complete protease inhibitor cocktail (Nacalai Tesque Inc.). Protein concentrations were measured using a Protein Assay Bicinchoninate Kit (Nacalai Tesque Inc.). Before the sodium dodecyl sulphate‐polyacrylamide gel electrophoresis (SDS‐PAGE), an aliquot of the extracted protein solution was mixed with equal volumes of the sample loading buffer containing 1% (vol/vol) 2‐mercaptoethanol, 4% (wt/vol) SDS, 125 mM of Tris–HCl (pH: 6.8), 10% (wt/vol) sucrose, and 0.01% (wt/vol) bromophenol blue. The mixture was then heated at 97°C for 3 min. Ten micrograms of protein were separated on an SDS‐polyacrylamide gel and electrically transferred to an Immuno‐Blot PVDF membrane (Bio‐Rad Laboratories). The blot was blocked by Blocking One (Nacalai Tesque Inc.) for 1 h at room temperature and incubated with primary antibodies overnight at 4°C in tris‐buffered saline containing 0.1% Tween‐20. Signals were detected using the ImmunoStar Zeta or LD (Wako Chemicals), quantified by C‐Digit (LI‐COR Biosciences), and expressed as arbitrary units. Coomassie Brilliant Blue (CBB) staining was used to verify consistent loading.

### 2.7 Primary antibodies for Western blotting

The following primary antibodies were used for the western blotting: anti‐p70S6K (#9202; Cell Signaling Technology), anti‐p‐p70S6K (Thr389, #9205; Cell Signaling Technology), anti‐rpS6 (#2217; Cell Signaling Technology), anti‐p‐S6 (Ser240/244, #5364P; Cell Signaling Technology), anti‐eIF4E‐binding protein 1 (4EBP1) (#9452; Cell Signaling Technology), anti‐p‐4EBP1 (Thr37/46, #2855S; Cell Signaling Technology), anti‐extracellular signal‐regulated kinase 1/2 (ERK1/2) (#9102; Cell Signaling Technology), anti‐p‐ERK1/2 (Thr202/Tyr204, #9101; Cell Signaling Technology), anti‐p38 MAPK (p38) (#9212; Cell Signaling Technology), and anti‐p‐p38 (Thr180/Tyr182, #9211; Cell Signaling Technology).

### 2.8 Muscle protein synthesis

Muscle protein synthesis was measured using the in vivo SUnSET (surface sensing if translation) method as described previously (Goodman et al., 2011). Under anesthesia, 0.04 μM puromycin/g body weight (FUJIFILM Wako Pure Chemical Co.) diluted in a 0.02 M PBS stock solution was injected in the mice intraperitoneally into the mice. The plantaris (using experiment 1) and gastrocnemius (using experiment 2) muscles were removed 15 min after puromycin administration. Following homogenization, as described above, and centrifugation at 3800 × *g* for 3 min at 4°C, the supernatant was collected and processed for western blotting. A mouse monoclonal anti‐puromycin antibody (Merck Millipore) was used to detect puromycin incorporation, which was determined as the sum of the intensities of all protein bans in the western blot.

### 2.9 Statistical analyses

Data are shown as means ± standard deviation. Two‐way analyses of variance were performed for all measurements. When a significant *p*‐value was obtained, statistical significance was calculated according to Tukey's method. The GraphPad Prism 7 software (GraphPad, Inc.) was used for all statistical calculations, and the significance level was set to *p* < 0.05 for all cases.

## 3 RESULTS

### 3.1 Experiment 1: Lactate administration during 2 weeks mechanical overload

**FIGURE 2:**
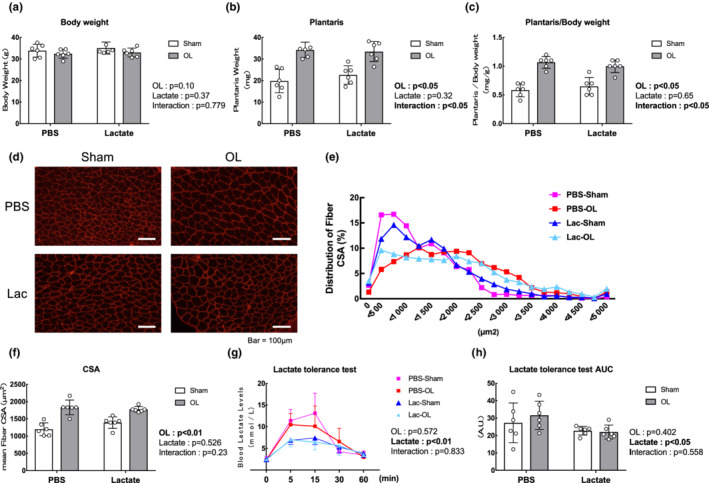
Lactate administration does not promote muscle hypertrophy following mechanical overload. (a) Body weight (*n* = 6 per group). (b and c) Changes in absolute and relative plantaris muscle weight (*n* = 6 per group). (d) Typical images of sarcolemma of the plantaris muscle stained by laminin alpha 2 antibody. (e) Distribution of fiber cross‐sectional area (CSA). The mean fiber size was obtained for each individual sample, followed by the calculating of the group data. (f) Mean fiber CSA. (g) Changes in lactate tolerance test. (h) Lactate tolerance test AUC. All data are expressed as means ± SD and individual values (*n* = 6). Significant differences were assessed via two‐way ANOVA followed by Tukey's multiple comparison test.

Figure 1 shows the study designs. To evaluate the effects of lactate administration on mechanical overload, we measured the animals' body weight and plantaris muscle wet weight. Body weight did not differ between the phosphate‐buffered saline (PBS) and lactate group (Figure 2a). The plantaris weight was significantly higher in the OL groups (main effect of OL: *p* < 0.05), but there was no difference between the PBS and lactate groups (Figure 2b,c). Laminin immunohistochemistry also revealed no difference in the gross morphology of muscle fibers between the PBS and lactate groups (Figure 2d). Consistent with the muscle weight data, the peak of the fiber cross‐sectional are (CSA) distribution was shifted rightward in the OL mice (Figure 2e), and the mean fiber CSA following OL was increased in the PBS and lactate‐administrated mice (main effect of OL: *p* < 0.05); however, there were no statistically significant effects from administering lactate (Figure 2f). In the lactate tolerance test, blood lactate levels and AUC were increased in PBS administrated mice compared with lactate administrated mice (main effect of Lactate: *p* < 0.05); however, there were no difference between Sham‐operated mice and OL‐treated mice (Figure 2g,h).

**FIGURE 3:**
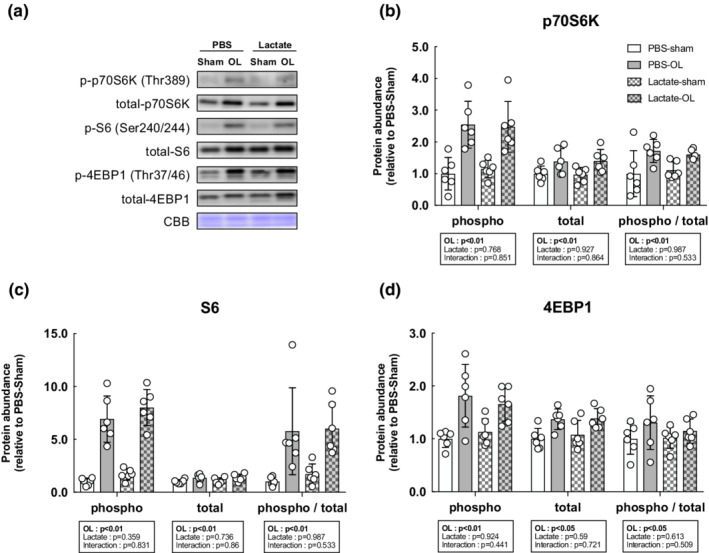
Lactate administration does not affect mTOR signaling activation following mechanical overload. (a) Representative western blot analyses. Coomassie brilliant blue (CBB) staining was used to verify consistent loading. (b) Total p70S6K. (c) Phosphorylation of p70S6K at Thr389. (d) Total S6, (e) Phosphorylation of S6 at Ser240/244. (f) Total 4EBP1. (g) Phosphorylation of 4EBP1 at Thr37/46. All data are expressed as means ± SD and individual values (*n* = 6). Significant differences were assessed via two‐way ANOVA followed by Tukey's multiple comparison test.

**FIGURE 4:**
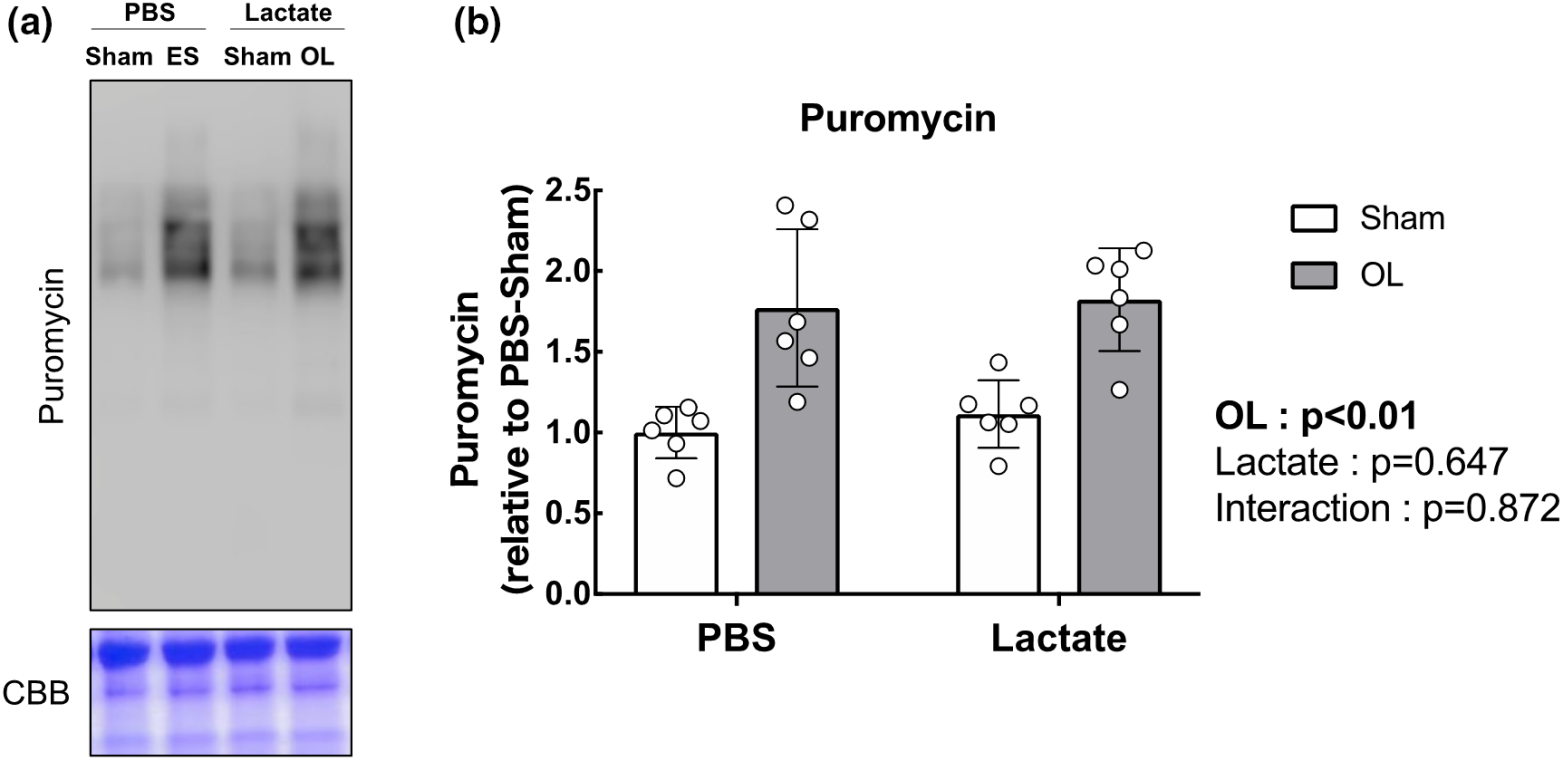
Lactate administration does not promote protein synthesis determined by quantification of the incorporative puromycin. (a) Representative image of western blot analysis. Coomassie brilliant blue (CBB) staining was used to verify consistent loading. (b) Protein synthesis in puromycin‐labeled protein (*n* = 6 in each group). All data are expressed as means ± SD and individual values (*n* = 6). Significant differences were assessed via two‐way ANOVA followed by Tukey's multiple comparison test.

**FIGURE 5:**
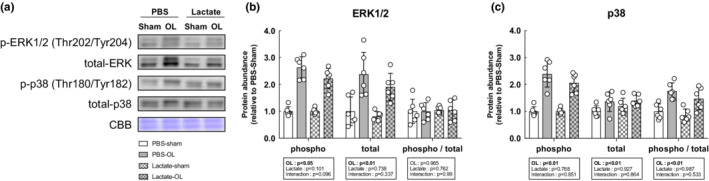
Lactate administration does not affect mechanical overload induced activation of downstream targets of mTOR independent signals. (a) Representative image of western blot analyses. (b) Total ERK1/2. Coomassie brilliant blue (CBB) staining was used to verify consistent loading. (c) Phosphorylation of ERK1/2 at Thr202//Tyr204. (d) Total p38. (e) Phosphorylation of p38 at Thr180/Tyr182. All data are expressed as means ± SD and individual values (*n* = 6). Significant differences were assessed via two‐way ANOVA followed by Tukey's multiple comparison test.

We next evaluated the activation status of the mTOR signaling pathway, known to play a central role in muscle growth through the activation of the downstream indicators p70S6K, S6, and 4EBP1. The total and phosphorylated protein levels of p70S6K, S6, and 4EBP1 were significantly increased following OL compared with the sham‐operated controls (main effect of OL: *p* < 0.05), but lactate administration did not affect the OL‐induced increase in mTOR signaling activation (Figure 3). In accordance with the results of the mTOR signaling pathway, muscle protein synthesis as measured by the SUnSET method was significantly increased following OL compared with the sham‐operated controls (main effect of OL: *p* < 0.05). However, no effects of lactate administration had no observable effect on the OL‐induced increase in protein synthesis (Figure 4). Furthermore, we investigated mitogen‐activated protein kinase (MAPK) signaling, which is known to be an upstream regulator of mTOR signaling and is activated under cellular stress, including mechanical stress. The total and phosphorylated protein levels of extracellular signal‐regulated kinase (ERK1/2) were both significantly increased following OL, while p38 phosphorylation was not altered. There was no effect of lactate on these protein levels (Figure 5).

### 3.2 Experiment 2: Effects of lactate administration by acute electrical stimulation

**FIGURE 6:**
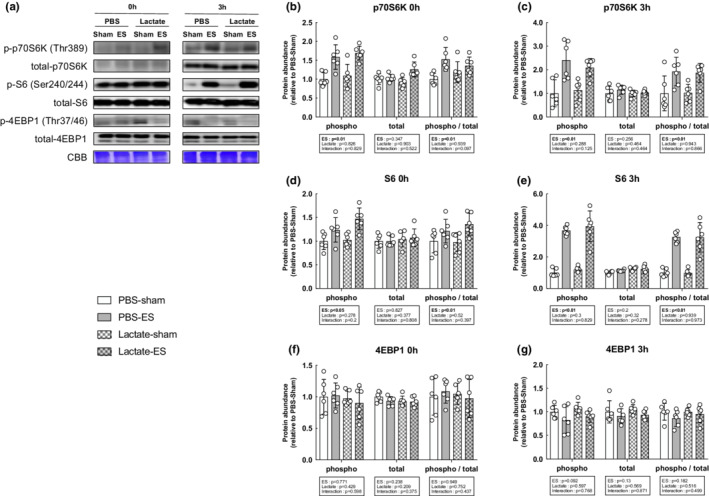
Lactate administration does not affect mTOR signaling activation at 0 or 3 h after acute resistance exercise. (a) Representative images of western blot analyses. Coomassie brilliant blue (CBB) staining was used to verify consistent loading. (b–g) The phosphorylation state of mTORC1‐dependent signaling pathways was determined and quantified using phospho‐specific antibodies. (b, c) Phosphorylation of p70S6K at Thr389. (d, e) Phosphorylation of S6 at Ser240/244. (f, g) Phosphorylation of 4EBP1 at Thr37/46. All data are expressed as means ± SD and individual values (*n* = 6). Significant differences were assessed via two‐way ANOVA followed by Tukey's multiple comparison test.

**FIGURE 7:**
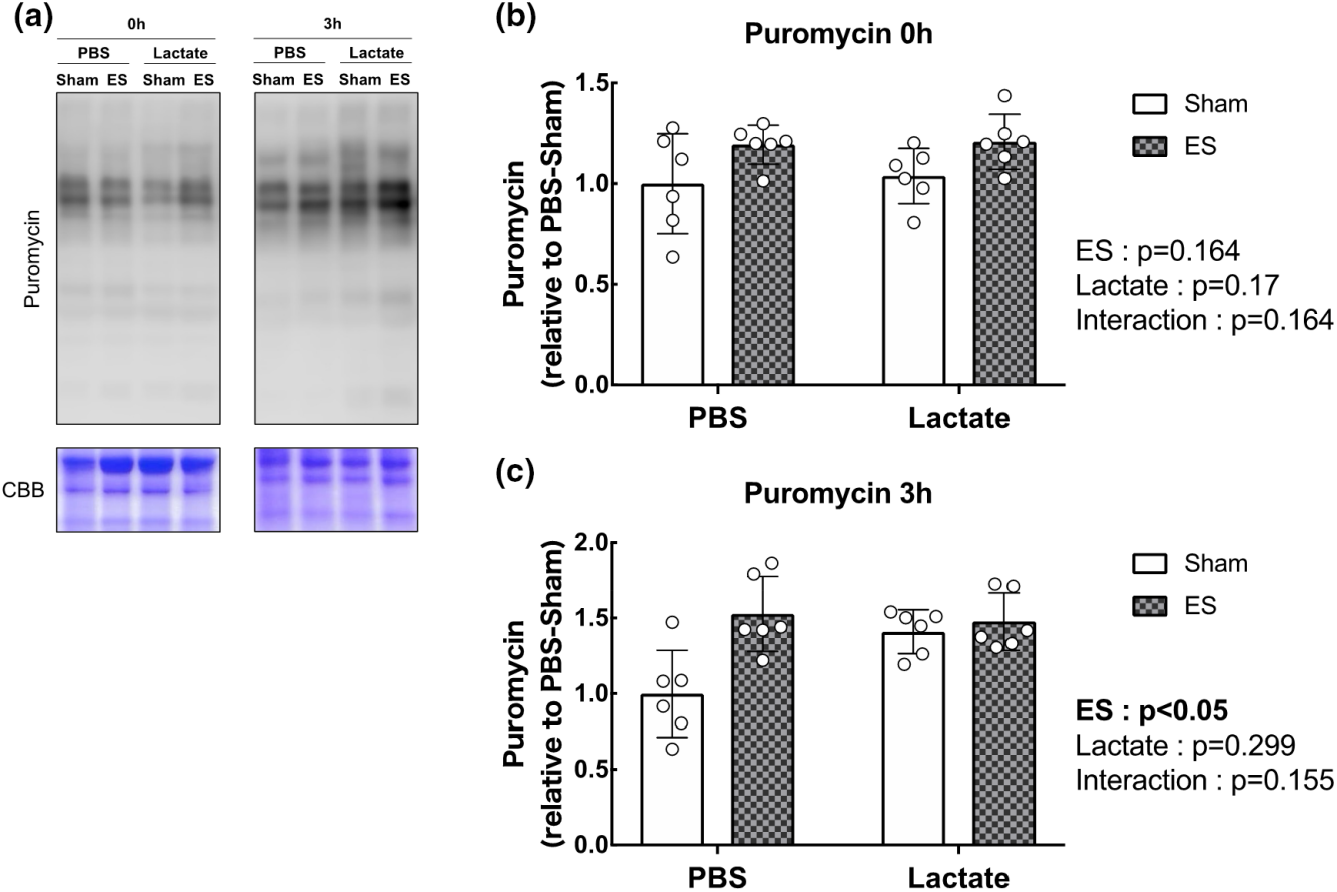
Lactate administration does not promote protein synthesis determined by quantification of the incorporative puromycin at 0 or 3 h after acute resistance exercise. (a) Representative image of western blot analysis. Coomassie brilliant blue (CBB) staining was used to verify consistent loading. (b, c) Protein synthesis in puromycin‐labeled protein at 0 and 3 h. all data are expressed as means ± SD and individual values (*n* = 6). Significant differences were assessed via two‐way ANOVA followed by Tukey's multiple comparison test.

**FIGURE 8:**
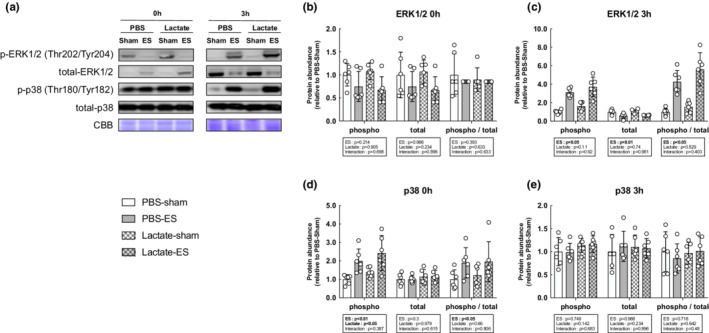
Effects of lactate administration on mTOR independent signals at 0 or 3 h after acute resistance exercise. (a) Representative images of western blot analyses. Coomassie brilliant blue (CBB) staining was used to verify consistent loading. (b–e) Phosphorylation and total state of mTORC1‐indeppendent signals were determined and quantified using phospho‐ and total specific antibodies. (b, c) Phosphorylation of ERK1/2 at Thr202//Tyr204, total and phospho/total ratio. (d, e) Phosphorylation of p38 at Thr180/Tyr182, total and phospho‐/total ratio. All data are expressed as means ± SD and individual values (*n* = 6). Significant differences were assessed via two‐way ANOVA followed by Tukey's multiple comparison test.

ES was applied when the blood lactate concentration reached 11.56 ± 1.2 mmol/L 15 min after lactate administrating. To evaluate the activation effects of lactate on mTOR signaling following acute ES, we examined p70S6K, S6, and 4EBP1 levels. The phosphorylated protein levels of p70S6K and S6 were significantly increased following ES compared with the sham controls at all time point (main effect of ES: *p* < 0.05), whereas the phosphorylation state of 4EBP1 was not altered. Lactate administration did not affect the ES‐induced increase in mTOR signaling activation (Figure 6). In line with mTOR signaling, ES‐induced increases in muscle protein synthesis at 3 h after ES; however, lactate administration did not affect these ES‐induced changes (Figure 7). Lastly, we further investigated MAPK signaling, and found that the phosphorylation state of ERK1/2 were significantly increased following ES compared with the sham controls at 3 h (main effect of ES: *p* < 0.05). The p38 phosphorylation levels were significantly increased immediately after ES (main effect of ES: *p* < 0.05) and significantly increased in the lactate‐administrated mice at this time point (main effect of lactate: *p* < 0.05) (Figure 8).

## 4 DISCUSSION

In this study, we investigated whether lactate administration augmented acute and chronic muscle hypertrophic responses, using well‐established rodent models. This in vivo model induces compensatory hypertrophy growth of the plantaris muscle through mechanical overload resulting from the surgical removal of the tendons from the synergist muscles (gastrocnemius and soleus), and is very useful as a model to observe exercise‐induced muscle hypertrophy and protein synthesis. Consistent with previous reports (Miyazaki et al., 2011; Moriya & Miyazaki, 2018; Shirai, Obara, & Takemasa, 2020; Uemichi et al., 2021), 14 days of mechanical overload induced 25%–50% muscle hypertrophy and enhanced protein synthesis with activated mTOR signaling in mouse skeletal muscle. Another rodent model of resistance exercise is high‐intensity muscle contraction; 4 weeks of training (three times a week) is shown to induce 10%–15% muscle hypertrophy (Kitaoka, Nakazato, & Ogasawara, 2016; Ogasawara et al., 2016). The ES model has the advantage of a high degree of control over muscle activity and loading distinct parameters compared to voluntary muscle activation models such as squat and ladder training. In addition, the voluntary muscle activation model is known to increase anabolic signaling activity, but muscle weight often does not increase after repeated exercise, probably due to insufficient exercise load or volume applied to the muscle (Adams & Bamman, 2012). We set post 0‐ and 3‐h time points for analyzing increased muscle protein synthesis to investigate the effect of lactate on acute resistance exercise‐induced protein synthesis. These time points were set in accordance with previous studies (Maruyama et al., 2020; Miyazaki et al., 2020). Using this mild model, we also found that acute high‐intensity muscle contractions increased mTOR signaling and muscle protein synthesis. However, we observed no effect of lactate administration on these indices in either model of muscle hypertrophy. In agreement with our results, a recent study reported that lactate does not affect the activation of muscle protein synthesis signaling associated with acute resistance exercise in humans, (Liegnell et al., 2020) suggesting that exogenous lactate administration does not affect exercise‐induced anabolic signaling in skeletal muscle.

There is growing evidence that lactate might serve as a signal for exercise‐induced adaptations. It has been demonstrated that single doses of lactate administration up‐regulate mRNA levels of genes related to oxidative metabolism in skeletal muscle (Hashimoto et al., 2007; Kitaoka, Takeda, et al., 2016), and that daily lactate doses increase muscle mitochondrial enzyme activity in mice (Takahashi et al., 2019; Takahashi et al., 2020). In vitro studies using skeletal muscle cells have shown that lactate activates the mTORC1 and ERK1/2 pathway, (Oishi et al., 2015) suggesting that lactate might have a positive effect on skeletal muscle mass. However, our results indicate that lactate does not enhance the activation of exercise‐induced protein synthesis signals and muscle hypertrophy. In human studies, resistance exercise increases the phosphorylation of 4EBP1 (Liegnell et al., 2020). However, animal studies using mice and rats reported that ES does not increase them (Ogasawara et al., 2020; Takegaki et al., 2019). The reason for this may be that in previous experiments, animal studies have used isometric contractions with local (gastrocnemius only) transcutaneous electrical stimulation. On the other hand, the phosphorylation and total protein of 4EBP1 is increased in the chronic model, mechanical overload. Therefore, we believe that if we can load the muscle by dynamic muscle contraction, we can reproduce the changes in 4EBP1.

OL is known to increase p38 (Carlson et al., 2001; Hornberger et al., 2005), but the change is triggered at a very early stage. Previous studies have shown that the increase in p38 is low at 24 h after overload. In our study, p38 protein expression increased immediately after ES, but decreased to basal level after 3 h. It is also known that lactate can alter p38. However, chronic lactate administration did not increase p38 (Takahashi et al., 2020). In human skeletal muscle, there is no difference in the phosphorylation state of p38 despite the fact that exercise‐induced PGC‐1α mRNA expression is increased when blood lactate levels increase during high‐intensity exercise (Percival et al., 2015). Furthermore, Hoshino et al reported that the exercise‐induced phosphorylation states of these kinases were not significantly altered by a decrease in lactate accumulation during high‐intensity exercise (Hoshino et al., 2015). Taken together, these kinases do not appear to be involved in the adaptation of hypertrophy induced by lactate. Therefore, we conclude that the increase in p38 was acute and there was no effect of p38 in promoting muscle hypertrophy.

Interestingly, previous studies have demonstrated that lactate enhances muscle regeneration and fiber hypertrophy in glycerol‐induced (Tsukamoto et al., 2018) and cardiotoxin‐injected (Ohno et al., 2019) regenerating mouse muscles. Similarly, we previously reported that lactate administration inhibited muscle mass loss under caloric restriction (Shirai et al., 2021). These observations suggest that lactate does not augment resistance exercise‐induced activation of molecular signaling but rather suppresses the decreases in muscle atrophy by activating mTOR signaling. Of note, lactate administration failed to prevent a denervation‐induced decline in muscle mass (Takahashi et al., 2021). We speculate that this failure was due to the compensatory activation of mTOR signaling in denervated muscles (Machida et al., 2012). Given that both mechanical overload and high‐intensity muscle contraction also activate mTOR signaling, our results indicate an absence of the additive effects of lactate administration in these muscle hypertrophy models.

The present study has limitation. Lactate administration into intraperitoneal space of mice is not the same as intracellular lactate production in humans during exercise. It is unclear in this study to what extent the lactate administration affected the muscle fibers during exercise. The dose of lactate we applied (1 g/kg of body weight) increased the blood lactate concentration to 11.56 ± 1.2 mmol/L. This amount is comparable to the amount of blood lactate increased by high‐intensity resistance exercise in humans (Mascher et al., 2008). However, it has been reported that some exercise protocols do not increase the lactate level to the present level (Liegnell et al., 2020), and it is possible that the dose administered in the present experiment is not consistent with the resistance exercise‐induced blood lactate concentration. On the other hand, although we did not measure the intramuscular lactate concentration in this study, it has been reported that the intramuscular concentration increases in human experiments (Liegnell et al., 2020), and it is possible that the intramuscular lactate concentration increased in this study as well.

In this study, we examined the effects of lactate administration on the activation of exercise‐induced protein synthesis signals and muscle hypertrophy using acute and chronic resistance exercise models. We found no effect of lactate on the acute or chronic exercise models with respect to hypertrophy and exercise‐induced mTORC1 or ERK signaling. Our results indicate that lactate does not affect exercise‐induced anabolic signaling properties in mouse skeletal muscle.

## AUTHOR CONTRIBUTIONS

T.S., Y.K., and K. Takeda. were conceived and designed this project; T.S., Y.K., K.U., and K. Tokinoya. performed the experiments; T.S. analyzed the data; T.S. prepared the figures; T.S., Y. K., and K.U. wrote paper; T.S., Y.K., K. Takeda., and T.T. revised manuscript. All authors read and approved the final manuscript.

## CONFLICT OF INTEREST

The authors declare that there are no conflicts of interest.
